# Comparing Perioperative Outcomes of Robotic-Assisted Versus Laparoscopic Liver Resection in Patients with Hepatocellular Carcinoma

**DOI:** 10.7150/jca.115543

**Published:** 2025-07-28

**Authors:** Xiao-Kun Huang, Lin-Lin Gan, Lei Liang, Kai Wang, Kai-Di Wang, Yang Yu, Zheng-Kang Fang, Yi Lu, Guo-Liang Shen, Dong-Sheng Huang, Cheng-Wu Zhang, Jun-Wei Liu, Jian Cheng

**Affiliations:** 1 General Surgery, Cancer Center, Department of Hepatobiliary & Pancreatic Surgery and Minimally Invasive Surgery, Zhejiang Provincial People's Hospital, Affiliated People's Hospital, Hangzhou Medical College, Hangzhou, Zhejiang, China.; 2 Department of Postgraduate Training Base Alliance of Wenzhou Medical University, Wenzhou, Zhejiang, China.; 3 Department of Hepatobiliary Surgery, Eastern Hepatobiliary Surgery Hospital, Second Military Medical University (Navy Medical University), Shanghai, China.; 4 Department of the Second School of Clinical Medicine, Zhejiang Chinese Medical University, Hangzhou, Zhejiang, China.; 5 Department of Urology, Shanghai Tenth People's Hospital, Tongji University School of Medicine, Shanghai, China.

**Keywords:** laparoscopy, robotic, liver resection, complication, comprehensive complication index

## Abstract

**Background**: Despite the implementation of laparoscopic and robotic-assisted liver resection (LLR vs. RLR) in many centers, there remains controversy surrounding the differences in perioperative outcomes between the two approaches. This study aims to clarify the discrepancies in perioperative outcomes between LLR and RLR through a prospective study.

**Methods:** Patients with HCC received LLR or RLR were included. The postoperative complications were categorized and evaluated employing the standardized Clavien-Dindo classification and the Comprehensive Complication Index (CCI) score. Specifically, the median CCI of 20.9 was set as the cut-off value for the occurrence of severe complications. A 1:2 propensity score matched (PSM) analysis was performed to control confounding bias.

**Results:** A total of 273 patients were included, of whom 213 (78%) patients received LLR and 60 (22%) patients received RLR. After PSM, RLR was associated with a longer operative time but shorter hospital stays (all P < 0.05). Postoperative outcomes in terms of overall complications, major and minor complications, and mortality were similar between RLR and LLR groups (all P > 0.05). Of note, RLR is significantly associated with a lower CCI score, especially server complications (OR 0.826, 95%CI 0.386-0.883, P = 0.023).

**Conclusions:** In terms of complication rates, RLR does not reduce the incidence of overall complications when compared to LLR, but it can reduce the severity of complications that occur. RLR, is a feasible and safe approach for patients with HCC.

## Introduction

Hepatocellular carcinoma (HCC) ranks as the sixth most prevalent primary malignancy and the fourth foremost contributor to cancer mortality globally^1^. Surgical intervention continues to occupy a paramount position in the curative therapy for patients with HCC. The advent of sophisticated technologies and highly maneuverable medical instruments in liver resection surgeries has ushered in a new era of minimally invasive procedures^2^. Notably, minimally invasive surgery for HCC has gained substantial clinical traction, surpassing traditional open surgery methods, owing primarily to its advantages such as diminished blood loss, mitigated postoperative discomfort, and accelerated post-hospitalization recovery periods^3^-^5^. Consequently, since its initial description in 1991^6^, laparoscopic liver resection (LLR) has emerged as a widely adopted therapeutic strategy for HCC. Furthermore, the report of the first robot-assisted liver resection (RLR) in 2003^7^, signified the dawn of robotic technology in minimally invasive hepatic surgery. Presently, both LLR and RLR have evolved into the standard operative procedures within the realm of minimally invasive liver resection, reflecting their widespread acceptance and clinical efficacy^8^, ^9^.

Over the past two decades, numerous studies have consolidated the safety profile and practicality of RLR^10^, ^11^. Nevertheless, the existence of disparities in the influence of RLR and LLR on patient outcomes, particularly in the realm of short-term prognosis, remains a contentious issue. In a seminal case-control study conducted by Berber et al. in 2010, a comparative analysis of RLR and LLR revealed no statistically significant variations in operative duration, blood loss, or the incidence of conversion to open surgery^12^. Traditional laparoscopic instrumentation, characterized by its two-dimensional visual field, restricted operative angles, and exacerbation of unintentional tremors, significantly heightens the complexity of surgical maneuvers, necessitating a protracted learning curve that impedes its widespread adoption in liver resection procedures^13^. Conversely, RLR addresses these limitations by offering a tenfold magnified, high-definition three-dimensional operative field, the dexterity akin to 'wrist-like' movements, and inherent tremor filtration for enhanced stability. This technology fosters superior hand-eye coordination and facilitates operations on ergonomic platforms, thereby enabling the execution of precise hepatectomies^14^. Despite the obvious technical advantages of robotics, there is no high-quality evidence that RLR is superior to LLR for safety and efficacy in patients with HCC. Recently, Sijberden et al. performed a multicenter retrospective study including 10,075 patients^15^. The results of the study demonstrated a statistically significant correlation between the application of RLR, as opposed to LLR, and an elevated percentage of textbook-defined favorable outcomes (78.3% vs. 71.8%, P < 0.001), concomitantly accompanied by a notably reduced incidence of overall morbidity (19.3% vs. 25.7%, P < 0.001). These results underscore the potential advantages of RLR over LLR in terms of enhancing the likelihood of achieving optimal treatment outcomes. However, the inclusion of complication variables in this study was not exhaustive, which may be attributed to its retrospective nature. In clinical practice, as the number of complication variables increases, the overall complication rate also tends to escalate, highlighting the limitation of using complication rates as the sole measure of outcomes^16^. Consequently, there is an urgent need for a more comprehensive indicator that can accurately reflect both the occurrence and severity of complications. To address this limitation, Slankamenac et al. developed a continuous scale called the comprehensive complication index (CCI)^17^. Therefore, the objective of this study is to evaluate potential differences in terms of perioperative outcomes of RLR and LLR in patients with HCC.

## Methods

### Patients

All patients who underwent curative surgical resection (R0) were pathologically confirmed to have ICC and were retrospectively enrolled from Jan. 2020 to Jan. 2024. R0 resection is defined as complete resection of the tumor with negative microscopic margins. The exclusion criteria were as follows: (1) age < 18, or > 80 years old, (2) received preoperative anti-infective or nutritional supportive treatments, (3) inflammatory diseases in the month before surgery (including acute pancreatitis or cholecystitis, pneumonia, etc.), (4) preoperative antitumor therapy, (5) previous history of abdominal surgery, (6) had concomitant other operations such as portal vein ligation, bile duct explorations, staged hepatectomy, gastrointestinal surgery or splenectomies, (7) had a conversion to open liver resection. All patients included in the study had obtained informed consent before surgery and agreed to have their data stored and used in the research. This study was consistent with the Declaration of Helsinki and approved by the Institutional Review Board at Hospital.

### Surgical procedures

All patients underwent preoperative multidisciplinary team discussion. The criteria for resectable HCC are based on liver function, tumor location, and future residual liver volume^18^, ^19^. The RLR was meticulously executed utilizing the Da Vinci Surgical System. During RLR, the hepatoduodenal ligament was routinely isolated and prepared for hepatic inflow occlusion, with the surgeon employing the intermittent Pringle maneuver judiciously, limiting each instance to no longer than 15 minutes, interspersed with 5-minute reperfusion intervals. The liver parenchyma transection was executed precisely using an ultrasonic scalpel, often complemented by intraoperative ultrasound or fluorescence guidance. Vascular and biliary structures were managed with precision, with smaller vessels and ducts secured through electric coagulation or titanium clips, while larger ones were clamped with sutures and plastic clips. The LLR, employing conventional laparoscopic tools, adheres to similar principles and operative steps as RLR. Both RLR and LLR were performed by experienced surgeons.

### Clinicopathological characteristics and perioperative morbidity

In the present study, all pertinent clinicopathological characteristics were retrospectively gathered from the electronic health records database, encompassing gender, age, American Society of Anesthesiologists (ASA) classification, Eastern Cooperative Oncology Group (ECOG) performance status, comorbid illness, etiology of liver disease, presence of cirrhosis, portal hypertension status, Child-Pugh class, preoperative serum levels of alanine aminotransferase (ALT) and aspartate aminotransferase (AST), alpha-fetoprotein (AFP) values, maximal tumor dimensions and count, as well as tumor location. During the surgical procedures, several intraoperative variables were documented, such as resection margin status, major hepatectomy (defined as resection of > 3 liver segments^20^), intraoperative blood loss, intraoperative blood transfusion, and duration of surgery. The time of surgery was excluded equipment preparation time. Concerning perioperative outcomes, the focus was on postoperative length of hospital stay, as well as 90-day mortality rates and 30-day morbidity. Comorbidities were comprehensively evaluated and categorized to include obesity, diabetes mellitus, chronic cardiovascular disease, obstructive pulmonary disease, and a history of renal dysfunction. The analysis also encompassed perioperative morbidity and mortality outcomes, specifically post-hepatectomy liver failure^21^, hemorrhage, blood transfusion, bile leakage, intra-abdominal infections, pneumonia, and the presence of pleural effusion or ascites. The duration of hospital stay was determined from the day of surgery until the date of discharge. Morbidity was further categorized into minor (Clavien-Dindo grades I-II)^22^ and major (Clavien-Dindo grades III-V) complications. Moreover, CCI scores were calculated for each patient by utilizing the online calculator (www.cci-calculator.com), which integrates all reported complications into the Clavien-Dindo classification system.

### Statistical analysis

Continuous data were reported as median (range) or mean (standard deviation), while categorical data were expressed as counts and percentages, as appropriately. The Mann-Whitney U test was used for continuous variables, Fisher's exact test, or Pearson's χ2 test was used for categorical variables to compare the difference between the two groups. To minimize potential selection bias, propensity score matching (PSM) was used to adjust for differences in baseline characteristics between the two groups. To assess the incidence of intraoperative and postoperative complications, all preoperative variables were included in the PSM analysis. PSM was performed using a 1:2 greedy, nearest neighbor matching algorithm without replacement, with a caliper of 0.01. To determine the true relationship between the surgical approach and the incidence of postoperative complications, univariate and multivariate logistic regression analyses were performed. A multivariate logistic regression model included variables that were significant in the univariate analyses (P < 0.1). A significance level of P < 0.05 was considered as statistical difference. Odds ratios (OR) and 95% confidence intervals (CI) were reported in the study. Statistical analysis was performed using R 4.3.1, and the required R packages include "haven", "MatchIt", "ggplot2" and "scitb".

## Results

### Baseline and tumor-related characteristics

A total of 273 patients diagnosed with HCC undergoing R0 resection were included, of whom 213 patients underwent LLR and 60 patients underwent RLR. The baseline and tumor-related characteristics of the patients who underwent RLR and LLR are shown in **Table 1**. Compared to the LLR group, patients who underwent RLR had a higher proportion of females (43.3% vs. 24.9%, P = 0.005). Meanwhile, patients with tumors in more complicated positions tend to choose RLR (P < 0.05). After 1:2 PSM with a caliper of 0.01, 159 patients (106 patients in the LLR group vs. 53 patients in the RLR group) were included for further analysis. Then, the results showed that there were no significant statistical differences in the distribution of all variables between the two groups (all P > 0.05).

### Comparisons of intraoperative and postoperative outcomes

A comparison of intraoperative and postoperative outcomes among patients who underwent RLR versus LLR in the overall cohort and the PSM cohorts are shown in **Table 2**. After PSM with a caliper of 0.01, the proportion of operation time greater than 180 min was higher in patients who underwent RLR, compared to LLR (58.5% vs. 41.5%, P = 0.032). The results also indicated that RLR had a lower proportion of intraoperative blood loss (LLR 20.8% vs. RLR 9.4%, P = 0.055) and blood transfusion (LLR 15.1% vs. RLR 7.5%, P = 0.135) than LLR, though there is no statistic difference. Importantly, no patients died within postoperative 90 days in the two groups. After PSM, the incidence of postoperative 30-day morbidity was 55.9% (LLR 56.6% vs. RLR 54.7%, P = 0.477). Of these, 42.7% was minor morbidity (LLR 41.5% vs. RLR 45.3%, P = 0.388) and 13.2% were major morbidity (LLR 15.1% vs. RLR 9.4%, P = 0.041). The median hospital stay after liver resection was 9 (range 7-15) days in the LLR group, and 6 (range 4-10) days in the RLR group (P = 0.015). The mean CCI of patients with complications in the LLR group was 15.6 (standard deviation 16.9), while that in the RLR group was 14.2 (standard deviation 13.3) (P = 0.025). The median CCI of 20.9, then, was set as the cut-off value for the occurrence of severe complications. The results showed that the incidence of severe complications in the RLR group was significantly lower than that in the LLR group (17.0% vs. 32.1%, P = 0.031).

### Independent risk factors of postoperative complications

Univariable and multivariable logistic regression analyses used to identify factors associated with postoperative complications and server complications are presented in **Table 3** and **Table 4**. The multivariable logistic regression analyses revealed that there was no significant difference in the incidence of postoperative overall complications between the RLR and LLR group (OR 0.892, 95%CI 0.624-1.176, P = 0.144). Of note, RLR can significantly reduce the incidence of server complications (OR 0.806, 95%CI: 0.386-0.883, P = 0.023). Meanwhile, comorbid illness, portal hypertension, tumor location, and major hepatectomy were also independent risk factors for both postoperative overall complications and server complications.

## Discussion

The present study compared the perioperative outcomes of RLR and LLR for patients with HCC, using propensity score matching, and identified several advantages of RLR over LLR. Although the results showed that RLR took longer operative time than LLR, a possible reason for this outcome is that we calculated the entire surgical duration, which may have included the setup time for the robotic equipment. Of note, the results showed that the median length of postoperative hospital stay in the RLR group was 3 days less than that in the LLR group. Furthermore, there was no significant difference in the overall complication rate between the RLR and LLR groups. However, the incidence of severe complications was significantly lower in the RLR group compared to the LLR group (OR 0.806, P = 0.023). In other words, compared to LLR, RLR can reduce the risk of severe complications by nearly 20%.

With the advancements in minimally invasive instrumentation, including laparoscopy and robotics, minimally invasive liver resection techniques have gained significant momentum^23^. Over the past three decades, LLR has garnered widespread acceptance among hepatic surgeons^24^. Recently, RLR has been introduced to address the inherent challenges of conventional laparoscopy, particularly in complex procedures involving the resection of tumors located in intricate regions such as the posterosuperior segments and demanding major hepatectomies^25^-^27^. RLR offers several technical advantages over LLR, including a three-dimensional operative view, enhanced camera stability, tremor elimination, and superior dexterity facilitated by the Endo wrist mechanism^28^. These advantages empower surgeons to perform intricate tissue dissections with unparalleled precision and control, potentially translating into optimized surgical outcomes^29^. While LLR has firmly established itself as a safe and viable surgical approach over the past decade, RLR remains an evolving technology^30^. The present study aims to delve deeper into the distinct differences between these two techniques, providing further clarity on their respective merits and implications for clinical practice.

The findings of previous studies demonstrated that RLR does not exhibit any inferiority compared to LLR concerning intraoperative blood loss. Chiow et al. conducted a multicenter retrospective analysis to compare the outcomes of RLR and LLR in the context of right posterior hepatic resection^30^. The results showed that RLR had lower rate of intraoperative blood loss and major complications. Hu et al. also performed a multicenter retrospective analysis to compare the outcomes of RLR and LLR in the context of minor liver resections of the anterolateral segments^31^. The results indicated that RLR showed lower median blood loss, decreased blood transfusion, lower open conversion and shorter post operative stay than LLR. Concurrently, a PSM analysis encompassing a comparison between 220 patients undergoing RLR and 769 patients subjected to LLR in the context of right and extended right liver resection unveiled no statistically discernible differences in terms of blood loss^32^, aligning with the findings of a separate, smaller, single-center study that similarly contrasted RLR and LLR outcomes^33^. In the present study, the results demonstrated that RLR had a lower proportion of intraoperative blood loss (LLR 20.8% vs. RLR 9.4%, P = 0.055) and blood transfusion (LLR 15.1% vs. RLR 7.5%, P = 0.135) than LLR, though there is no statistic difference. The potential reason may be that this study included relatively simple surgeries, including some left lateral lobe surgeries, which are not the type of cases that would highlight the advantages of robotic surgery. However, the results of this study show that RLR significantly shortened the postoperative hospital stays compared to LLR, which is consistent with previous findings.

In addition to comparing intraoperative variables, clinicians are also concerned about the incidence of postoperative complication rates between the RLR and LLR. Many studies showed there was no statistically significant difference in postoperative complications^30^, ^34^, ^35^. The present study also indicated that, compared to LLR, RLR did obviously increased the risk of the overall complications (OR 0.892, P = 0.144). One possible reason for this finding is that more complication-related variables were taken into account, particularly including patients with underlying diseases. Furthermore, clinicians not only focus on the differences in the overall incidence of complications, but also pay more attention to the differences in the occurrence of postoperative severe complications between the two surgical approaches. Although previous studies have reported that RLR has a lower incidence of major complications compared to LLR, due to the small sample size, a multivariate regression analysis could not be performed to confirm whether RLR is an independent protective factor for reducing postoperative major complications. Additionally, since the Clavien-Dindo classification only considers the grade of the patient's most severe complication, it does not fully reflect the overall occurrence of complications in the patient. Therefore, this study employed a new complication scoring variable, the CCI score. The CCI score is a novel method for quantifying surgical complications, taking into account both the severity and number of complications. It is calculated by integrating information on the Clavien-Dindo classification of complications, adjusting for their severity and clustering within patients. In the present study, the results showed that the incidence of severe complications was significantly lower in the RLR group compared to the LLR group (OR 0.806, P = 0.023).

There are also some limitations in this study. Firstly, despite our adoption of a prospective study design and PSM, there is still a potential for selection bias. Secondly, in this study, we comprehensively enrolled patients undergoing all types of liver surgeries, without any targeted selection towards those with complex procedures. Despite the reported advantages of RLR in surgeries for liver cancer located in specific regions, we contend that it also offers certain benefits in general liver surgeries. Consequently, we included all patients based on their individual clinical scenarios, ensuring a comprehensive representation. Thirdly, the utilization of the robotic surgical platform stands as a pivotal aspect deserving meticulous consideration. While laparoscopy is commonly accessible upon request, the robotic system is frequently shared among various surgical specialties, thereby potentially constraining its availability. Moreover, the choice of operative modality was ultimately entrusted to the patients themselves, with the substantial cost associated with robotic procedures significantly influencing their decision-making process. Nonetheless, the present findings suggest that the employment of robotic techniques is likely advantageous, particularly in meticulously selected scenarios. Further research is necessary to provide more evidence in support of this. To reduce the impact of surgical learning curve, all the patients included in the study were operated by senior attending doctors with high qualifications, and the past laparoscopic HCC resection surgeries exceeded 200 cases. Meanwhile, for the learning curve of robotic surgery, all the surgeons had more than 50 cases of HCC resection experience.

## Conclusion

The results of our study indicate that RLR is a safe and feasible minimally invasive approach with comparable efficacy to LLR. In terms of complication rates, RLR does not reduce the incidence of overall complications when compared to LLR, but it can reduce the severity of complications that occur.

## Figures and Tables

**Table 1:** Comparison baseline characteristics between robotic-assisted and laparoscopic liver resection in patients with hepatocellular carcinoma.

Variables (N, %)	Before PSM			After PSM		
	LLR (n = 213)	RLR (n = 60)	P	LLR (n =106)	RLR (n = 53)	P
Baseline characteristics						
Sex, female/male	53 (24.9)/ 160 (75.1)	26 (43.3)/ 34 (56.7)	0.005	38 (35.8)/ 68 (64.2)	21 (39.6)/ 32 (60.4)	0.384
Age, > 60/ ≤ 60 years	141 (66.2)/ 72 (33.8)	33 (55.0)/ 27 (45.0)	0.111	64 (60.4)/ 42 (39.6)	29 (54.7)/ 24 (45.3)	0.304
ASA score, > 2/ ≤ 2	61 (28.6)/ 152 (71.4)	22 (36.7)/ 38 (63.3)	0.232	31 (29.2)/ 75 (70.8)	18 (34.0)/ 35 (66.0)	0.333
Performance status, ≥ 1/ 0-1	61 (28.6)/ 152 (71.4)	18 (30.0)/ 42 (70.0)	0.477	29 (27.4)/ 77 (72.6)	16 (30.2)/ 37 (69.8)	0.423
Comorbid illness, with/without	71 (33.3)/ 142 (66.4)	18 (30.0)/ 42 (70.0)	0.374	38 (35.8)/ 68 (64.2)	15 (28.3)/ 38 (71.7)	0.377
Etiology of liver disease, HBV/ Others	174 (81.7)/ 39 (18.3)	51 (85.0)/ 9 (15.0)	0.351	91 (85.8)/ 15 (14.2)	45 (84.9)/ 8 (15.1)	1.000
Cirrhosis, with/without	137 (64.3)/ 76 (35.7)	43 (71.7)/ 17 (28.3)	0.289	79 (74.5)/ 27 (25.5)	37 (69.8)/ 16 (30.2)	0.572
Portal hypertension, with/without	55 (25.8)/ 158 (74.2)	12 (20.0)/ 48 (80.0)	0.227	30 (28.3)/ 76 (71.7)	12 (22.6)/ 41 (77.4)	0.567
Child-Pugh grade, A/B	187 (87.8)/ 26 (12.2)	55 (91.7)/ 5 (8.3)	0.404	100 (94.3)/ 6 (5.7)	49 (92.5)/ 4 (7.5)	0.732
Preoperative ALT level, > 40/ ≤ 40 U/L	34 (16.0)/ 179 (84.0)	9 (15.0)/ 51 (85.0)	0.518	19 (17.9)/ 84 (82.1)	9 (17.0)/ 44 (83.0)	1.000
Preoperative AST level, > 40/ ≤ 40 U/L	61 (28.6)/ 152 (71.4)	14 (23.3)/ 46 (76.7)	0.261	35 (33.0)/ 71 (67.0)	14 (26.4)/ 39 (73.6)	0.468
Tumor-related variables						
AFP, > 20/ ≤ 20 ng/L	150 (70.4)/ 63 (29.6)	40 (66.7)/ 20 (33.3)	0.341	71 (67.0)/ 35 (33.0)	37 (69.8)/ 16 (20.2)	0.857
Maximum tumor size, > 5/ ≤ 5 cm	71 (33.3)/ 142 (66.7)	21 (35.0)/ 39 (65.0)	0.462	40 (37.7)/ 66 (62.3)	18 (34.0)/ 35 (66.0)	0.728
Tumor numbers, Multiple/ Single	39 (18.3)/ 174 (81.7)	6 (10.0)/ 54 (90.0)	0.125	18 (17.0)/ 88 (83.0)	6 (11.3)/ 46 (88.7)	0.482
Tumor location			0.001			0.953
Left hepatic lobe	102 (47.9)	13 (21.7)		28 (26.4)	13 (24.5)	
Right hepatic lobe	88 (41.3)	33 (55.0)		53 (50.0)	29 (54.7)	
Median hepatic lobe	17 (8.0)	11 (18.3)		23 (21.7)	10 (18.9)	
Caudate lobe	6 (2.8)	3 (5.0)		2 (1.9)	1 (1.9)	

LLR, laparoscopic liver resection; RLR, robotic-assisted liver resection; ASA, American Society of Anesthesiologists; HBV, hepatitis B virus; ALT, alanine aminotransferase; AST, aspartate transaminase; AFP alpha-fetoprotein; PSM, propensity score matched.

**Table 2:** Comparison of intraoperative and perioperative outcomes between robotic-assisted and laparoscopic liver resection in patients with hepatocellular carcinoma.

Variables (N, %)	Before PSM			After PSM		
	LLR (n = 213)	RLR (n = 60)	P	LLR (n = 106)	RLR (n = 53)	P
Intraoperative outcomes						
Resection margin, < 1/ ≥ 1 cm	101 (47.4)/ 112 (52.6)	23 (38.3)/ 37 (61.7)	0.135	37 (34.9)/ 59 (65.1)	20 (37.7)/ 33 (62.3)	0.428
Major hepatectomy, ≥ 3/ < 3 segments	76 (35.7)/ 137 (64.3)	24 (40.0)/ 36 (60.0)	0.320	46 (43.4)/ 60 (56.6)	22 (41.5)/ 31 (58.5)	0.479
Intraoperative blood loss, > 600/ ≤ 600 ml	55 (25.8)/ 158 (74.2)	13 (21.7)/ 47 (78.3)	0.317	22 (20.8)/ 84 (79.2)	5 (9.4)/ 48 (90.6)	0.055
Intraoperative blood transfusion, with/without	28 (13.1)/ 185 (86.9)	6 (10.0)/ 54 (90.0)	0.515	16 (15.1)/ 90 (84.9)	4 (7.5)/ 49 (92.5)	0.135
Operation time, > 180/ ≤ 180 min	73 (34.3)/ 140 (65.7)	34 (56.7)/ 26 (43.3)	0.002	44 (41.5)/ 62 (58.5)	31 (58.5)/ 22 (41.5)	0.032
Perioperative outcomes						
Postoperative hospital stays*	10 (7, 22)	8 (4, 17)	0.727	9 (7, 15)	6 (4, 10)	0.015
Postoperative hospital stays, ≥7d	124 (58.2)	29 (48.3)	0.112	65 (61.3)	23 (43.4)	0.024
Hospitalization expenses* ($)						
Postoperative 90-day mortality	0 (0)	0 (0)	NE	0 (0)	0 (0)	NE
Postoperative 30-day morbidity	124 (58.2)	31 (51.7)	0.224	60 (56.6)	29 (54.7)	0.477
liver failure	37 (16.0)	9 (15.0)	0.518	20 (18.9)	8 (15.1)	0.362
postoperative bleeding	17 (8.0)	4 (6.7)	0.493	9 (8.5)	4 (7.5)	0.552
postoperative blood transfusion	26 (12.2)	11 (18.3)	0.156	13 (12.3)	7 (13.2)	0.525
bile leakage	44 (20.7)	14 (23.3)	0.387	23 (21.7)	11 (20.8)	0.532
intra-abdominal infection	29 (13.6)	8 (8.3)	0.193	19 (17.9)	5 (9.4)	0.118
pneumonia	45 (21.1)	8 (13.3)	0.120	29 (27.4)	7 (13.2)	0.032
pleural effusion	54 (25.4)	9 (15.0)	0.062	19 (17.9)	8 (15.1)	0.418
ascites	39 (18.3)	6 (10.0)	0.087	18 (17.1)	6 (11.3)	0.244
Minor (Clavien-Dindo I-II)	95 (44.6)	25 (41.7)	0.400	44 (41.5)	24 (45.3)	0.388
Major (Clavien-Dindo III-V)	29 (13.6)	6 (10.0)	0.310	16 (15.1)	5 (9.4)	0.041
CCI scores^#^	27.3 ±12.8	19.7 ±8.3	0.019	15.6 ±16.9	14.2 ±13.3	0.025
CCI scores, > 20.9	68 (31.9)	12 (20.0)	0.049	34 (32.1)	9 (17.0)	0.031

LLR, laparoscopic liver resection; RLR, robotic-assisted liver resection; PSM, propensity score matched; NE, not evaluable; CCI: Comprehensive Complication Index; *median (range); #mean ± standard deviation.

**Table 3:** Univariable and multivariable logistic analysis of risk factors associated with postoperative overall complications for patients with hepatocellular carcinoma.

Variables	UV OR (95% CI)	P	MV OR (95% CI)	P
Sex, male vs. female	1.087 (0.601-1.965)	0.782		
Age, > 60 vs. ≤ 60 years	1.224 (0.697-2.150)	0.495		
ASA score, > 2 vs. ≤ 2	1.165 (0.636-2.134)	0.621		
Performance status, ≥2 vs. 1	1.059 (0.595-1.884)	0.845		
Comorbid illness, yes vs. no	2.609 (1.028-6.224)	0.044	1.305 (1.013-2.328)	0.038
HBV, yes vs no	1.184 (0.642-2.182)	0.589		
Cirrhosis, yes vs. no	1.247 (0.678-2.293)	0.477		
Portal hypertension, yes vs. no	1.668 (1.072-4.141)	0.027	1.260 (1.059-1.904)	0.045
Child-Pugh grade, B vs. A	1.268 (0.672-4.141)	0.260		
AST level, > 40 vs. ≤ 40 U/L	1.483 (0.831-2.010)	0.530		
ALT level, > 40 vs. ≤ 40 U/L	0.983 (0.508-1.900)	0.959		
AFP level, > 20 vs. ≤ 20 ug/L	0.993 (0.549-1.796)	0.982		
Tumor size, > 5 vs. ≤ 5 cm	1.155 (1.058-1.900)	0.015	NS	
Tumor number, ≥ 2 vs. 1	1.890 (0.837-2.818)	0.747		
Tumor location, Left hepatic lobe	Ref.		Ref.	
Right hepatic lobe	1.363 (1.045-2.440)	0.034	1.147 (1.019-1.331)	0.022
Median hepatic lobe	1.508 (1.094-4.914)	0.021	1.206 (1.058-3.176)	0.005
Caudate lobe	1.624 (1.009-2.409)	0.019	1.556 (1.013-2.358)	0.002
Resection margin, ≥ 1 vs. < 1cm	1.202 (0.708-2.043)	0.496		
Major hepatectomy, yes vs. no	1.136 (1.024-2.281)	0.029	2.135 (1.245-3.661)	0.006
Blood loss, > 600 vs. ≤ 600 mL	1.112 (0.524-2.360)	0.782		
Blood transfusion, yes vs. no	1.334 (0.499-3.565)	0.566		
Operation time, ≥ 180 vs. < 180 min	2.015 (1.102-3.683)	0.023	NS	
Type of resection, RLR vs. LLR	0.801 (0.450-0.963)	0.038	0.892 (0.624-1.176)	0.144

ASA, American Society of Anesthesiologists; HBV, hepatitis B virus; ALT, alanine aminotransferase; AST, aspartate transaminase; AFP alpha-fetoprotein; LLR, laparoscopic liver resection; RLR, robotic-assisted liver resection; MV, multivariable; UV, univariable; OR, odds ratio; CI, confidence interval; NS, no significance.

**Table 4:** Univariable and multivariable logistic analysis of risk factors associated with comprehensive complication index for patients with hepatocellular carcinoma.

Variables	UV OR (95% CI)	P	MV OR (95% CI)	P
Sex, male vs. female	1.56 (0.778-2.914)	0.224		
Age, > 60 vs. ≤ 60 years	1.657 (0.860-3.193)	0.132		
ASA score, > 2 vs. ≤ 2	1.114 (0.568-2.184)	0.753		
Performance status, ≥2 vs. 1	1.183 (0.593-1.553)	0.484		
Comorbid illness, yes vs. no	1.353 (1.072-2.726)	0.037	1.341 (1.016-2.512)	0.009
HBV, yes vs no	1.066 (0.812-1.424)	0.295		
Cirrhosis, yes vs. no	1.157 (0.579-2.312)	0.680		
Portal hypertension, yes vs. no	2.420 (1.085-6.616)	0.005	1.228 (1.059-2.138)	0.012
Child-Pugh grade, B vs. A	2.336 (1.066-5.653)	0.006	NS	
AST level, > 40 vs. ≤ 40 U/L	1.064 (0.505-2.238)	0.871		
ALT level, > 40 vs. ≤ 40 U/L	1.904 (0.756-4.797)	0.172		
AFP level, > 20 vs. ≤ 20 ug/L	1.237 (0.618-2.478)	0.548		
Tumor size, > 5 vs. ≤ 5 cm	1.525 (1.046-1.924)	0.038	NS	
Tumor number, ≥ 2 vs. 1	1.156 (0.743-1.651)	0.483		
Tumor location, Left hepatic lobe	Ref.			
Right hepatic lobe	1.145 (1.057-2.368)	0.045	1.087 (1.024-1.856)	0.026
Median hepatic lobe	1.282 (1.023-2.669)	0.014	1.154 (1.019-1.958)	0.018
Caudate lobe	1.512 (1.184-7.224)	0.008	1.462 (1.105-3.023)	0.002
Resection margin, ≥ 1 vs. < 1cm	1.621 (0.592-2.946)	0.113		
Major hepatectomy, yes vs. no	1.794 (1.225-4.374)	0.003	1.266 (1.018-3.517)	0.001
Blood loss, > 600 vs. ≤ 600 mL	2.012 (0.505-3.311)	0.078	NS	
Blood transfusion, yes vs. no	1.293 (0.505-5.144)	0.593		
Operation time, ≥ 180 vs. < 180 min	1.644 (0.559-5.144)	0.164		
Type of resection, RLR vs. LLR	0.773 (0.361-0.883)	0.025	0.806 (0.386-0.883)	0.023

ASA, American Society of Anesthesiologists; HBV, hepatitis B virus; ALT, alanine aminotransferase; AST, aspartate transaminase; AFP alpha-fetoprotein; LLR, laparoscopic liver resection; RLR, robotic-assisted liver resection; MV, multivariable; UV, univariable; OR, odds ratio; CI, confidence interval; NS, no significance.

## Abbreviations

HCC: hepatocellular carcinoma
LLR: laparoscopic liver resection
RLR: robotic-assisted liver resection
ASA: American Society of Anesthesiologists
HBV: hepatitis B virus
ALT: alanine aminotransferase
AST: aspartate transaminase
AFP: alpha-fetoprotein
CCI: Comprehensive Complication Index
PSM: propensity score matched
OR: odds ratio
CI: confidence interval
